# Diagnostic Performance of Different Simulated Low-Dose Levels in Patients with Suspected Cervical Abscess Using a Third-Generation Dual-Source CT Scanner

**DOI:** 10.3390/diagnostics10121072

**Published:** 2020-12-10

**Authors:** Moritz T. Winkelmann, Saif Afat, Sven S. Walter, Eva Stock, Vincent Schwarze, Andreas Brendlin, Manuel Kolb, Christoph P. Artzner, Ahmed E. Othman

**Affiliations:** 1 Department for Diagnostic and Interventional Radiology, University Hospital Tuebingen, 72076 Tuebingen, Germany; Moritz.Winkelmann@med.uni-tuebingen.de (M.T.W.); Sven.Walter@med.uni-tuebingen.de (S.S.W.); eva.stock@gmx.net (E.S.); andreas.brendlin@med.uni-tuebingen.de (A.B.); manuel.kolb@med.uni-tuebingen.de (M.K.); christoph.artzner@med.uni-tuebingen.de (C.P.A.); ahmed.e.othman@googlemail.com (A.E.O.); 2 Department of Radiology, University Hospital LMU, 81337 Munich, Germany; vincent.schwarze@med.uni-muenchen.de

**Keywords:** cervical abscess, neck computed tomography, radiation dosage, iterative reconstruction, third-generation dual-source CT, image reconstruction, simulated dose levels

## Abstract

The aim of this study was to investigate the effects of dose reduction on diagnostic accuracy and image quality of cervical computed tomography (CT) in patients with suspected cervical abscess. Forty-eight patients (mean age 45.5 years) received a CT for suspected cervical abscess. Low-dose CT (LDCT) datasets with 25%, 50%, and 75% of the original dose were generated with a realistic simulation. The image data were reconstructed with filtered back projection (FBP) and with advanced modeled iterative reconstruction (ADMIRE) (strengths 3 and 5). A five-point Likert scale was used to assess subjective image quality and diagnostic confidence. The signal-to-noise ratio (SNR) of the sternocleidomastoid muscle and submandibular gland and the contrast-to-noise ratio (CNR) of the sternocleidomastoid muscle and submandibular glandular fat were calculated to assess the objective image quality. Diagnostic accuracy was calculated for LDCT using the original dose as the reference standard. The prevalence of cervical abscesses was high (72.9%) in the cohort; the mean effective dose for all 48 scans was 1.8 ± 0.8 mSv. Sternocleidomastoid and submandibular SNR and sternocleidomastoid muscle fat and submandibular gland fat CNR increased with higher doses and were significantly higher for ADMIRE compared to FBP, with the best results in ADMIRE 5 (all *p* < 0.001). Subjective image quality was highest for ADMIRE 5 at 75% and lowest for FBP at 25% of the original dose (*p* < 0.001). Diagnostic confidence was highest for ADMIRE 5 at 75% and lowest for FBP at 25% (*p* < 0.001). Patient-based diagnostic accuracy was high for all LDCT datasets, down to 25% for ADMIRE 3 and 5 (sensitivity: 100%; specificity: 100%) and lower for FBP at 25% dose reduction (sensitivity: 88.6–94.3%; specificity: 92.3–100%). The use of a modern dual-source CT of the third generation and iterative reconstruction allows a reduction in the radiation dose to 25% (0.5 mSv) of the original dose with the same diagnostic accuracy for the assessment of neck abscesses.

## 1. Introduction

Cervical abscesses often occur as a complication of infection due to different pathogens such as *Streptococcus viridans*, *Staphylococcus epidermidis*, and *Staphylococcus aureus*. The most common causes are pharyngotonsillitis, dental infection, or intravenous drug abuse [1,2]. To localize the primary extent of infection and to exclude possible complications, contrast-enhanced multi-slice computed tomography is used as the primary diagnostic tool in the clinical work-up [3,4]. Typical complications of a cervical abscess include mediastinitis and jugular vein thrombosis [5]. To diagnose a cervical abscess and potential complications, the CT scan has to include the region between the base of the skull and the aortic arch [6]. Cervical abscesses can occur at any age, but the highest incidence is found in patients between 14 and 30 years of age [2,7]. The use of computed tomography results in a considerable exposure to ionizing radiation with a generally increasing cumulative radiation dose in the population, which may increase the risk of radiation-induced carcinogenesis [8,9]. To ensure imaging is undertaken according to according to the safety principle ALARA (as low as reasonably achievable), strategies to reduce radiation dose for patients are necessary. Various technical approaches for dose reduction have been developed, including modulation of tube current, automatic selection of tube voltage, reduced kVp settings, and reduction of *z*-axis scan coverage [10,11,12]. Lowering the radiation dose with standard filter back projection (FBP) will result in increased noise levels of the images with decreasing diagnostic confidence [13]. Iterative reconstruction has been shown to compensate for a lower dose by significantly reducing image noise while maintaining diagnostic confidence [11,13].

Dual-source CT scanners (DSCT) of the third generation allow further dose reduction and a reduction in image noise while maintaining image quality by using an integrated circuit detector system (Stellar Infinity, Siemens) for tube voltage selection and an improved iterative reconstruction algorithm [14,15]. The potential benefits of applying the advanced modeled iterative reconstruction technique (ADMIRE) on a DSCT for dose reduction in terms of image quality are well known [10,13]. ADMIRE, which can be applied in various strengths, performs noise reduction at the raw data level and can adjust a variety of image-degrading effects [16,17]. However, it has not yet been investigated what influence dose reduction with ADMIRE compared to FBP has on the diagnostic accuracy for the detection of neck abscesses at different dose levels.

Thus, the aim of this study was to evaluate image quality and diagnostic accuracy at different radiation doses in contrast-enhanced neck CT using third-generation DSCT and the different reconstruction modes FBP and ADMIRE.

## 2. Materials and Methods

**Figure 1:**
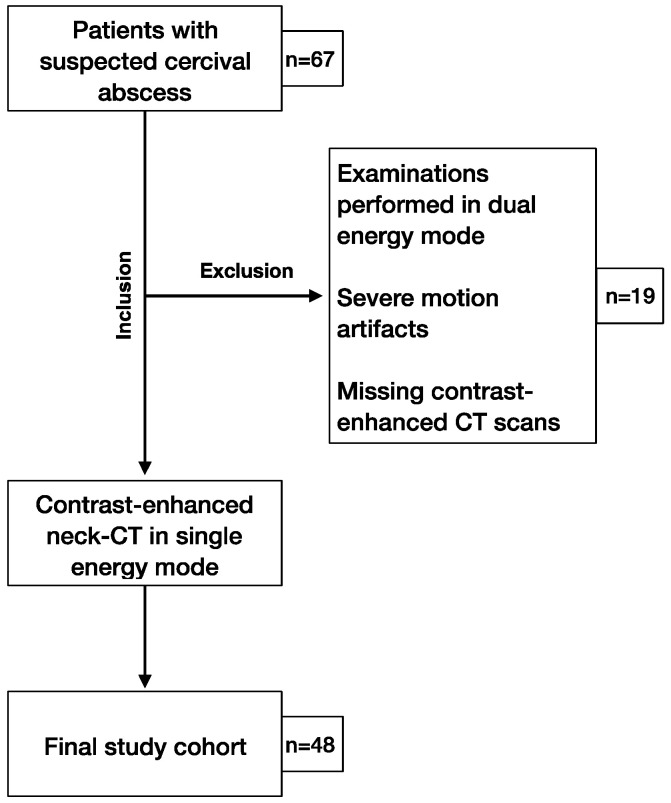
Study flowchart of patient inclusion.

The local institutional review board approved this retrospective analysis of patient data (approval code: 735/2018BO2, approval date: 1 April 2019) To avoid selection bias, only patients from routine clinical work were included in the study. No screening by age, sex, weight, or other characteristics was performed. Retrospectively, raw datasets of 48 patients with clinical suspicion of cervical abscesses were generated using realistic simulation software (ReconCT, Siemens Healthineers, Erlangen, Germany). Between September 2018 and February 2019, all patients who were referred to the radiology department with suspeced cervical abscess were included in this study. Exclusion criteria were CT examinations without contrast medium or performed in dual energy mode and severe motion artefacts (Figure 1).

### 2.1. Imaging Protocol

All neck CT examinations were performed with iodine contrast medium (Iomeron 400, Bracco, Konstanz, Germany) at a flow rate of 1.5 mL/s using an automated double syringe power injector (Medrad, Bayer, Leverkusen, Germany). All investigations were performed on a third-generation dual-source CT scanner with 2 × 192 slices (Somatom Force, Siemens Healthineers, Forchheim, Germany): single-energy mode with activated automatic attenuation-based tube-current modulation for exposure control (CareDose4D); automatic kV selection (CareKV); gantry rotation, 0.25 s; pitch, 0.7; collimation, 0.6 × 40 × 2 mm (with *z*-flying focal spot). A double bolus was administered as standard for optimal soft tissue contrast: image acquisition 3 min after the first bolus (70 mL) and 30 s after the second bolus (20 mL), followed by a saline flush. The CT scans were performed at a reference tube voltage of 120 kVp and reference tube current–time product of 200 mAs. All images were reconstructed with ADMIRE strength 2, Bv40d kernel, 3 mm thick sections, and 3 mm section increments.

### 2.2. Low-Dose Simulation

The proprietary reconstruction platform ReconCT (version 14.2.0.40998, Siemens Healthineers, Forchheim, Germany) was used to simulate the raw datasets of the CT examinations. It has already been shown in several studies that it is possible to simulate CT examinations with reduced mAs and, thus, lower doses using ReconCT [17,18,19]. Low-dose datasets were generated using three different dose levels: 25%, 50%, and 75% of the original dose. For each dose level, one dataset was reconstructed using FBP and ADMIRE strength 3 and 5. All images were reconstructed with Bv40d kernel, 3 mm thick sections, and 3 mm section increments.

### 2.3. Radiation Dose

Volumetric CT dose index (CTDIvol) in terms of the 32 cm dosimetry phantom, dose–length product (DLP), and effective tube current–time product were drawn from the patient’s protocol to determine radiation exposure from the original CT datasets. The dose values of the original datasets in our study corresponded to the approved diagnostic reference levels (CTDIvol: 15 mGy; DLP: 330 mGy·cm) of the local authorities responsible for radiation protection [20]. The total effective dose was calculated using a commercially available, web-based dose management platform (Radimetrics Enterprise Platform, Version 2.6 b, Bayer Healthcare, Leverkusen, Germany).

### 2.4. Subjective Image Analysis

Image analysis was performed on a dedicated workstation (syngo.via, A30A; Siemens Healthineers, Germany) by three independent radiologists with 3, 4, and 5 years of experience in head and neck imaging, who were blinded to the dose levels and reconstruction algorithms, as well as all patient information. In order to avoid recall bias, the cases were anonymized, randomized, and interpreted in multiple sessions with a 6 week interval. Image quality and diagnostic confidence were evaluated independently by all three readers using a five-point Likert scale: 1, very poor; 2, poor; 3, moderate; 4, good; 5, very good. Subjective image quality regarding sharpness, noise, and artefacts was evaluated by two readers (Reader 1, Reader 2) using a five-point Likert scale: 1, major; 2, substantial; 3, moderate; 4, minor; 5, none. Moderate, good/minor, and very good/none were defined as diagnostically acceptable. Since diagnostic accuracy was the main focus of this study, three blinded readers evaluated the images for the presence or absence of a neck abscess.

### 2.5. Objective Image Analysis

All measurements were performed on a commercially available Picture Archiving and Communication System (PACS) workstation. Circular regions of interests (ROIs)with a constant size of 200 mm^2^ were plotted in the sternocleidomastoid muscle, cervical fat, and submandibular gland. Inclusion of adjacent anatomical structures or focal regions with inhomogeneities was avoided. Signal attenuation and image noise were measured in Hounsfield units (HU). Averaged values were calculated from three measurements to ensure data consistency. Image noise was quantified as standard deviation (SD) of each measured anatomical structure. The signal-to-noise ratio (SNR) was calculated for the sternocleidomastoid muscle and submandibular gland using the following formula:

(1) $$\mathrm{SNR}=\frac{\mathrm{Mean}\;\mathrm{signal}\;\mathrm{intenisty}}{\mathrm{SD}}$$

The sternocleidomastoidal muscle fat contrast-to-noise ratio and the submandibular glandular fat contrast-to-noise ratio were calculated as previously described in the literature [21] as follows:

(2) $$\mathrm{CNR}=\frac{\mathrm{HU}\left(\mathrm{Soft}-\mathrm{Tissue}\;\mathrm{Structure}\right)-\mathrm{HU}\left(\mathrm{Fat}\right)\;}{\mathrm{Image}\;\mathrm{Noise}\left(\mathrm{Fat}\right)}$$

### 2.6. Reference Standard

To verify diagnostic accuracy, the original standard-dose CT datasets were reviewed as the reference standard for all three readers, as well as by a senior radiologist (blinded) with 8 years of experience. In addition to the images with the original dose, the clinical report was also used to verify the accuracy of the diagnosis. Cases were classified as negative if the senior radiologist did not detect any neck abscess in the original 100% radiation dose datasets, and the final clinical report confirmed this.

### 2.7. Statistics

The available data were analyzed using SPSS (SPSS Statistics 26, IBM Corp., Armonk, New York, NY, USA). Continuous variables are presented as the mean ± standard deviation, and relative frequencies are presented as *n* (%). The Wilcoxon pairing test was used to calculate the differences in image quality and diagnostic confidence between different dose levels and reconstruction modes. Quantitative image analysis was evaluated by using repeated measures of ANOVA. Intraclass correlation (ICC) was used to calculate the interrater agreement. ICC values ≤0.5 were defined as poor, those 0.51–0.75 were defined as moderate, those 0.76–0.90 were defined as good, and those >0.90 were defined as excellent consistency. Crosstables with 95% confidence intervals were evaluated to calculate sensitivity, specificity, negative predicted value (NPV), and positive predicted value (PPV). A *p*-value < 0.05 was considered statistically significant.

## 3. Results

### 3.1. General Results

The final population that encountered the inclusion criteria was based on 48 patients (age: 45.5 ± 17.7, range 17 ± 83; male: 33, female: 15), all of whom were included in the final analysis (Table 1). In the final report, 35 patients were diagnosed with cervical abscess, of whom 28 had a peritonsillar abscess and seven had a parapharyngeal abscess. Other pathologies such as parotitis (*n* = 4), tonsillitis (*n* = 3), lymphadenitis colli (*n* = 3), pharyngitis (*n* = 1), sialadenitis (*n* = 1), and odontogenic inflammation (*n* = 1) were detected in a total of 12 patients. In one patient, no pathology causing the described symptoms was found.

### 3.2. Radiation Dose

The original datasets had a mean CTDIvol of 5.7 ± 1.3 mGy. The mean dose–length product was 184.7 ± 67.5 mGy·cm and mean scan length was 32.7 ± 8.9 cm. The resulting mean effective dose calculated was 1.8 ± 0.8 mSv. The clinical dose did not exceed the permitted local diagnostic reference values for neck CT [20]. The mean effective tube current–time product was 278.9 ± 90.8 mAs (range: 115–457 mAs), and the mean effective tube voltage was 81.7 ± 10.2 kV (range: 70–120 kV).

### 3.3. Subjective Image Analysis

**Figure 2:**
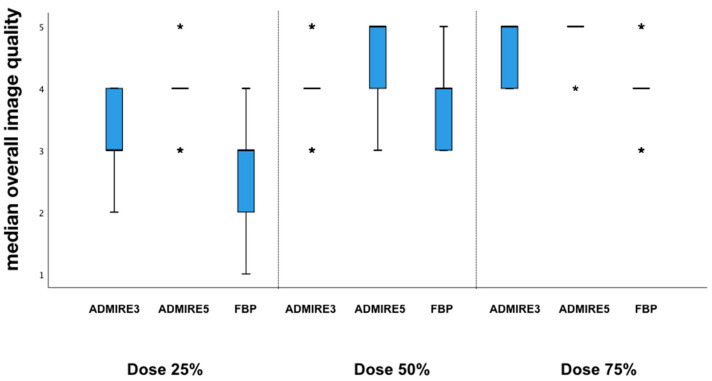
Overall subjective image quality. Given is the median of advanced modeled iterative reconstruction (ADMIRE) 3, ADMIRE 5, and filtered back projection (FBP) at different dose levels.

**Figure 3:**
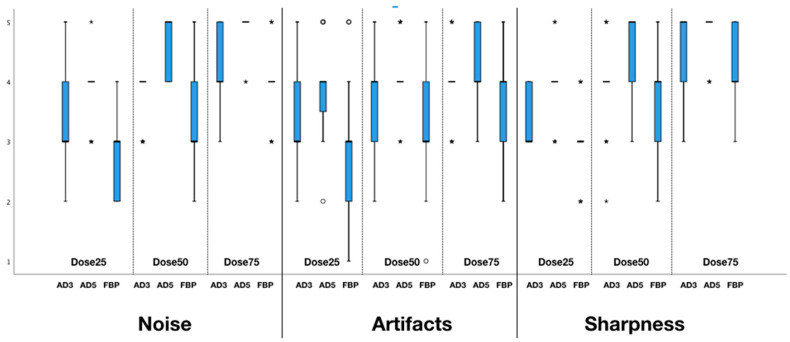
Artifacts, sharpness, and noise at different dose levels and reconstructions: Given is the median of ADMIRE 3, ADMIRE 5, and FBP.

Subjective overall image quality increased significantly for all reconstructions for higher doses (Figure 2 and Figure 3). ADMIRE 5 had significantly superior image quality at all dose levels of 25–75% of the original dose compared to ADMIRE 3 and FBP. ADMIRE 3 (mean (interquartile range): 3 (3–4)) and ADMIRE 5 (4 (4–4)) demonstrated better image quality with a dose reduction of 25% of the original dose than FBP (3 (3–3)). ADMIRE, in general, showed significantly better image quality than FBP at all dose levels between 25% and 75% of the original dose. At a dose reduction of 50% of the original dose, ADMIRE 5 (5 (5–5)) had the best results compared to ADMIRE 3 (4 (4–4)) and FBP (4 (4–4)) (all *p* < 0.001). For a visualization of the results, refer to Figure 2.

ADMIRE 5 (4 (4–4)) demonstrated the lowest image noise compared to ADMIRE 3 (3 (3–4)) and FBP (3 (3–3)) at a dose reduction of 25% of the original dose. Iterative reconstructed images with ADMIRE 3 (4 (4–4)) and ADMIRE 5 (5 (5–5)) also showed significantly lower noise than FBP (3 (3–4)) at a dose reduction of 50%. Similar differences were observed at 75% of the original dose for ADMIRE 3 (4 (4–5)), ADMIRE 5 (5 (5–5)), and FBP (4 (4–5)) (all *p* < 0.001).

In terms of artefacts, differences were observed among ADMIRE 3 (3 (3–4)), ADMIRE 5 (4 (4–4)), and FBP (3 (2–3)) at a dose reduction of 25% of the original dose, as well as for 50% (AD3: 4 (4–4); AD5: 4 (4–4); FBP: 3 (3–4)) and 75% of the original dose (AD3: 4 (4–4); AD5: 4 (4–5); FBP: 4 (4–4)). Sharpness was significantly higher for ADMIRE 5 (4 (4–4)) than for ADMIRE 3 (3 (3–4)) and FBP (3 (3–4)) at a dose reduction of 25% of the original dose. Similar differences between the reconstructions modes were observed at 50% (AD3: 4 (4–4); AD5: 5 (5–5); FBP: 4 (4–4)) and 75% (AD3: 5 (5–5); AD5: 5 (5–5); FBP: 4 (4–5)) of the original dose (all *p* < 0.001) (Figure 3).

Concerning diagnostic confidence, iteratively reconstructed images with ADMIRE 3 (4 (4–5)) and ADMIRE 5 (4 (4–5)) showed higher values compared to FBP (4 (4–4)) at 25% of the original dose (*p* < 0.001). However, very good diagnostic confidence was observed for all datasets at dose reductions of 50% and 75% of the original dose (5 (5–5)).

**Figure 4:**
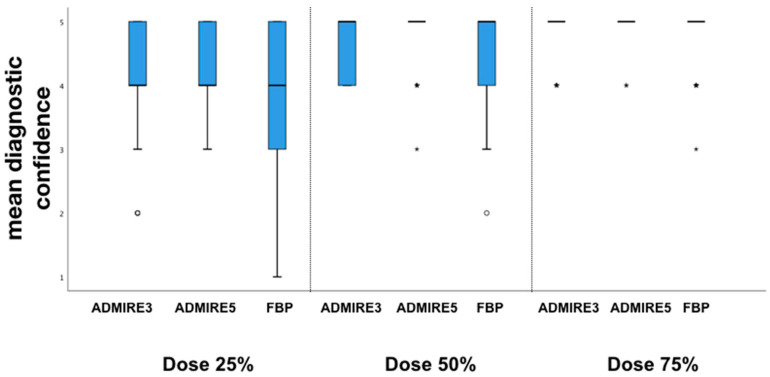
Mean diagnostic confidence. Box-and-whisker plots at different dose levels of overall diagnostic confidence in detection of cervical abscess.

Diagnostic confidence for all reconstructions and dose levels is shown in Figure 4. Interrater agreement was good for overall image quality with an ICC of 0.79 (confidence interval (CI) 95%: 0.71–0.87) and excellent for diagnostic confidence with 0.92 (CI 95%: 0.88–0.95).

### 3.4. Objective Image Analysis

The means and standard deviations of SNR and CNR are summarized in Table 2.

ANOVA with repeated measures for SNR of the sternocleidomastoid (violation of sphericity, Greenhouse–Geisser correction, Mauchly-W (65) < 0.001, *p* < 0.001) showed SNR values to significantly depend on method of reconstruction and dose level (F(3.718,147.769) = 106.354, *p* < 0.001, partial η² = 0.694).

Likewise, due to violation of sphericity after Greenhouse–Geisser-corrected ANOVA, SNR values of the submandibular gland (Mauchly-W (65) < 0.001, *p* < 0.001) indicated significant interactions for different reconstruction methods and dose levels (F(2.490,117.052) = 47.280, *p* < 0.001, partial η^2^ = 0.501).

ANOVA with repeated measurements for CNR of the sternocleidomastoid muscle fat (violation of sphericity, Greenhouse–Geisser correction, Mauchly-W (65) < 0.001, *p* < 0.001) again showed a significant dependence of CNR values to different modes of reconstruction and radiation dose levels (F(3.088,145.123) = 56.891, *p* < 0.001, partial η^2^ = 0.548).

Regarding CNR of the mandibular gland fat, Greenhouse–Geisser-corrected ANOVA with repeated measurements (Mauchly-W (65) < 0.001, *p* < 0.001) likewise showed CNR values to depend significantly on the method of reconstruction and the dose levels (F (3.065, 144.041) = 81.546, *p* < 0.001, partial η^2^ = 0.634).

Bonferroni-corrected post hoc tests showed SNR for the sternocleidomastoid muscle and submandibular gland to increase significantly with dose (*p* < 0.001). Additionally, SNR was higher in patients with ADMIRE reconstruction than in FBP reconstructed images (*p* < 0.001). ADMIRE 5 had a higher SNR than ADMIRE 3 at all dose levels (*p* < 0.001). Sternocleidomastoid muscle fat and submanibular gland fat CNR increased significantly with dose increase (*p* < 0.001). Compared to FBP, images reconstructed with ADMIRE had significantly higher CNR at all dose levels (*p* < 0.001). ADMIRE 5 had a significantly higher CNR than ADMIRE 3 at all dose levels (*p* < 0.001).

### 3.5. Diagnostic Accuracy

**Figure 5:**
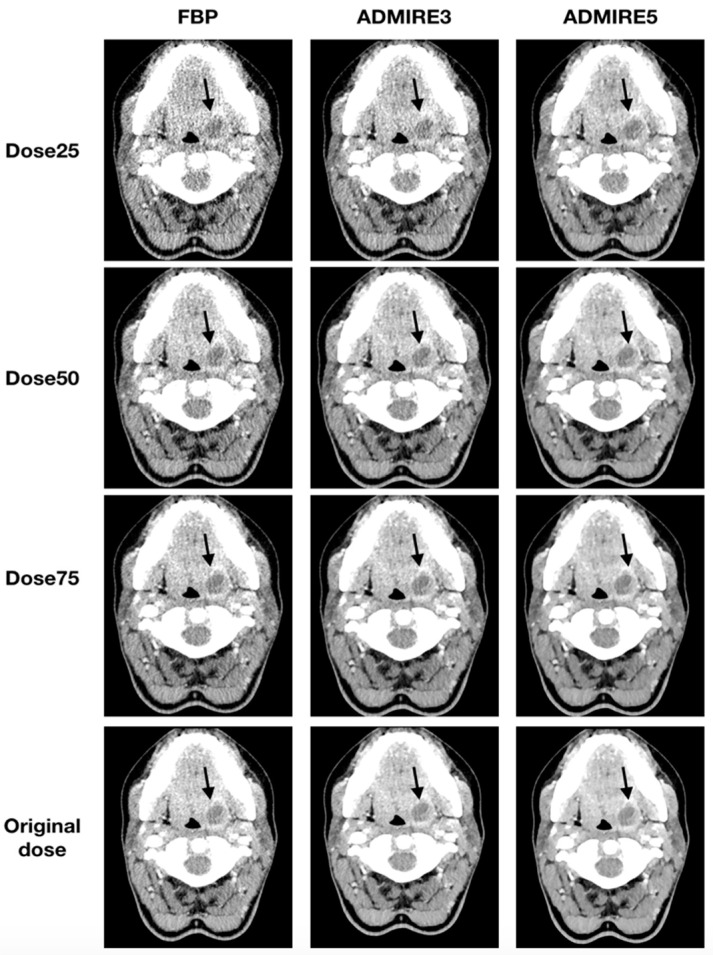
Axial tomographic slices of an 18 year old female patient with left-sided peritonsillar abscess (arrow). The images were reconstructed with FBP, ADMIRE 3, and ADMIRE 5 (from left to right) at 75%, 50%, and 25% dose reduction of the original dose.

**Figure 6:**
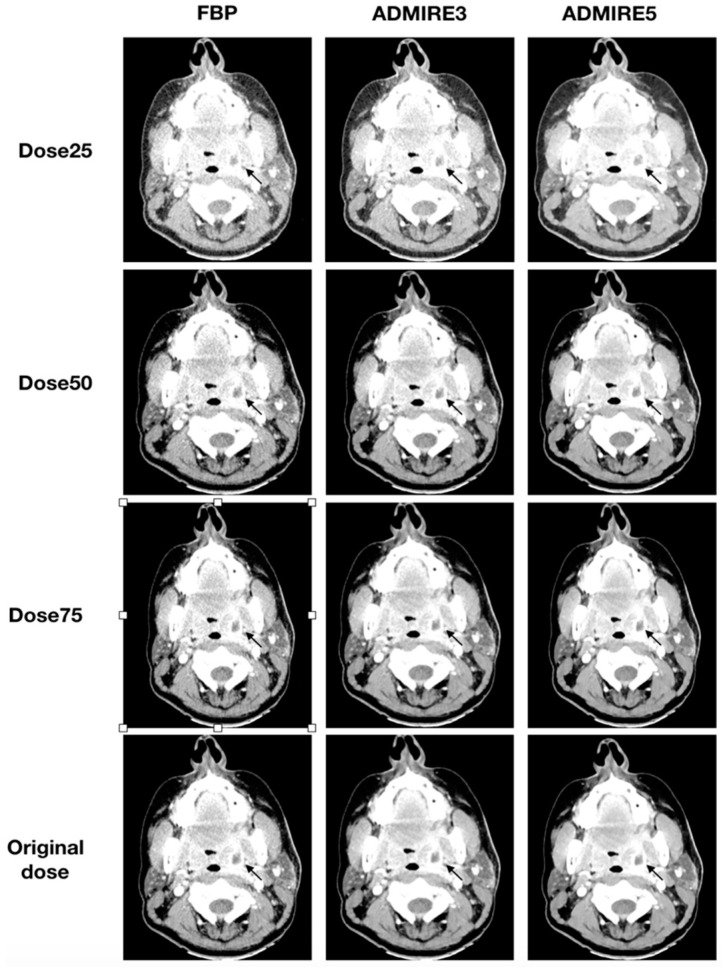
Axial tomographic slices of a 46 year old female patient with left-sided peritonsillar abscess (arrow). All images were reconstructed with FBP, ADMIRE 3, and ADMIRE 5 (from left to right) at 75%, 50%, and 25% dose reduction of the original dose. Reader 1 missed the abscess in reconstruction mode FBP at a dose of 25%.

All 48 patients were analyzed for the presence of a neck abscess. A total of 35 abscesses were diagnosed in the original datasets. All three blinded readers had a sensitivity of 100% (95% CI: 88–100) and specificity of 100% (95% CI: 72–100) for all simulated low-dose datasets, which were iteratively reconstructed with ADMIRE 3 or ADMIRE 5. FBP datasets had a sensitivity of 100% (95% CI: 88–100) and a specificity of 100% (95% CI: 72–100) for a dose reduction to 50% of the original dose. Deviations were only found in FBP reconstructed datasets at a dose of 25% of the original dose. Illustrations of the different reconstructions at the respective dose levels are shown in Figure 5 and Figure 6.

For FBP datasets at a dose reduction of 25% of the original dose, Reader 1 had a sensitivity of 88.6% (95% CI: 72–96) and a specificity of 92.3 (95% CI: 62–99). Ratings revealed one false-positive case and four false-negative cases, resulting in a positive predictive value (PPV) of 96.9% (95% CI: 82–99) and a negative predictive value (NPV) of 75% (95% CI: 47–91). Reader 2 had three false-negative cases and no false-positive case, resulting in a sensitivity of 91.4% (95% CI: 76–98) and a specificity of 100% (95% CI: 72–100) and a PPV of 100% (95% CI: 87–100) and NPV of 81.3% (95% CI: 54–95). Reader 3 had two false-negative cases and no false-positive case, resulting in a sensitivity of 94.3% (95% CI: 80–99) and a specificity of 100% (95% CI: 72–100) and a PPV of 100% (95% CI: 87–100) and NPV of 86.7% (95% CI: 58–98). Results for FBP at a dose reduction of 25% of the original dose are shown in Table 3.

Interrater agreement was excellent (ICC: 1.0) for all datasets except FBP at a dose reduction of 25% of the original dose. The interrater agreement for these datasets was still nearly perfect, with an ICC of 0.86–0.95.

## 4. Discussion

In this study, we evaluated the effect of simulated dose reduction on image quality and diagnostic performance in patients with suspected neck abscesses. Our results show that, by means of advanced modeled iterative reconstruction (ADMIRE), a dose reduction to up to 25% of the original dose is possible while maintaining diagnostic accuracy and acceptable image quality.

Our results are relevant because a large proportion of patients with cervical abscesses are young adults. Thus, a dose-reduced examination in these patients would be desirable [12,22]. Previous studies have already shown that a reduction in the scan area or a lowering of the tube voltage can result in a substantial dose reduction in patients with suspected cervical abscesses [10,12]. Another option for dose reduction in modern CT scanners is the use of iterative reconstruction, which has been shown in previous studies to have the potential to reduce the dose while maintaining good image quality [11,13,23]. To the best of our knowledge, no study has yet examined diagnostic performance in addition to image quality on low-dose datasets reconstructed with advanced modeled iterative reconstruction. Scholtz et al. [10] demonstrated that, by simultaneously lowering the tube voltage and using iterative reconstruction (ADMIRE) on a third-generation DSCT compared to FBP on a second-generation DSCT, a dose reduction of the head and neck region of up to 36% is possible. In comparison, this study was able to demonstrate that good image quality is possible with ADMIRE on a third-generation DSCT up to 50% dose reduction and that acceptable image quality is still present at 25% of the total dose. Furthermore, this work showed that, up to 50% dose reduction, all neck abscesses using both ADMIRE strengths and FBP were equally well detected by all three readers. This is consistent with our observations in subjective image quality, including noise and sharpness, where up to 50% dose reduction was measured as good to excellent for both ADMIRE strengths and as good to acceptable for FBP. However, with a dose reduction to 25% of the original dose, FBP achieved significantly inferior values in both image quality and diagnostic accuracy. The objective image quality parameters had their peak with ADMIRE 5 at all dose levels, while ADMIRE 3 had lower values but performed significantly better compared to FBP.

While a dose reduction of 25% of the original dose was observed for ADMIRE 3 and 5 with a sensitivity and specificity of 100%, the sensitivity and specificity for FBP with a dose reduction of 25% of the original dose were 88.6–94.3% and 92.3–100%, respectively. It is well known that the use of ADMIRE leads to a significant reduction in noise compared to FBP, resulting in better soft-tissue contrast, which may improve the diagnosis of neck abscesses, especially in patients with a suspected abscess in the head and neck region, where good soft-tissue contrast is particularly important for diagnosis [11,24,25,26]. In terms of overall image quality, noise, artefacts, and sharpness, we observed that ADMIRE 5 was superior to ADMIRE 3, especially at lower doses. Multiple studies demonstrated that an increase in ADMIRE strength leads to lower noise and, thus, higher soft-tissue contrast in abdominal CT [27,28]. As our results suggest, these observations may be transferable to other aspects of image quality, such as artefacts and sharpness. Regarding diagnostic accuracy, however, there were no differences between the two ADMIRE strengths used in our study. Thus, it would be interesting to see how these perform at even lower dose levels below 25% of the original dose.

Our study has limitations. First, our results are based on simulated raw datasets instead of neck CT scans with different tube voltages. These were not performed in order to avoid excessive radiation doses. However, the applied simulation tool is an accurate and robust method for generating low-dose CT scans [19,29]. Furthermore, we used a retrospective study design with a relatively small number of patients. By evaluating the image data intraindividually with three different readers, we tried to reduce possible bias. Due to a large amount of reconstructed image data, we used multiple reading sessions in intervals of 6 weeks to reduce recall bias and reader fatigue.

In conclusion, the use of a modern dual-source CT of the third generation and iterative reconstruction allows for a dose reduction to 25% of the original dose (0.5 mSv) while maintaining diagnostic accuracy and acceptable image quality for the assessment of neck abscesses.

## Figures and Tables

**Table 1:** Patient characteristics. M, male; F, female.

Variables	n (%)/Mean ± SD
Patients	48
Age (years) Sex (M/F)	45.5 ± 17.7 33 (68.8%)/15 (31.2%)
Clinical diagnosis	Cervical abscess (*n* = 35) Peritonsillar (*n* = 28) Parapharyngeal (*n* = 7) Other pathologies (*n* = 12) Parotitis (*n* = 4) Tonsillitis (*n* = 3) Lymphadenitis colli (*n* = 2) Pharyngitis (*n* = 1) Sialadenitis (*n* = 1) Odontogenic inflammation (*n* = 1) No pathology (*n* = 1)

**Table 2:** Signal-to-noise ratio (SNR) and contrast-to-noise ratio (CNR) calculations.

		Dose25			Dose50			Dose75	
	AD3	AD5	FBP	AD3	AD5	FBP	AD3	AD5	FBP
**Signal-to-noise ratio**									
Sternocleidomastoid muscle Submandibular gland	4.4 ±1.7 5.7 ± 2.4	5.9 ± 2.3 7.5 ± 2.9	3.0 ± 1.0 4.5 ± 1.6	5.5 ± 1.9 6.8 ± 2.6	7.3 ± 2.9 8.6 ± 3.5	4.0 ± 1.4 5.3 ± 1.9	6.3 ± 2.4 7.5 ± 2.9	8.4 ± 3.2 9.3 ± 4.3	4.7 ± 1.7 5.9 ± 2.2
**Contrast-to-noise ratio**									
Sternocleidomastoid muscle fat Submandibular gland fat	7.9 ± 3.5 9.4 ± 4.6	11.1 ± 4.9 13.1 ± 6.4	6.1 ± 2.2 7.3 ± 3.0	10.4 ± 3.3 12.2 ± 4.2	14.6 ± 8.3 16.9 ± 9.1	7.9 ± 2.5 9.2 ± 3.3	12.3 ± 4.6 14.5 ± 6.0	15.7 ± 5.9 18 ± 7.8	9.3 ± 2.9 10.9 ± 3.9

Dose 25, 50, and 75 indicates the percentage of the original dose values; AD3 = ADMIRE 3; AD5 = ADMIRE 5; FBP = filtered back projection.

**Table 3:** Diagnostic accuracy of low-dose computed tomography (LDCT) datasets for the detection of cervical abscesses.

Dose (Recon Mode) and Reader 1–3	Sensitivity % (95% CI)	Specificity % (95% CI)	PPV % (95% CI)	NPV % (95% CI)
Dose 25				
AD3, AD5	100 (88–100)	100 (88–100)	100 (73–100)	100 (87–100)
FBP:				
Reader 1	88.6 (72–96)	92.3 (62–99)	96.9 (82–99)	75 (47–91)
Reader 2	91.4 (76–98)	100 (72–100)	100 (87–100)	81.3 (54–95)
Reader 3	94.3 (80–99)	100 (72–100)	100 (87–100)	86.7 (58–98)
Dose 50				
AD3, AD5, FBP				
Reader 1–3	100 (88–100)	100 (72–100)	100 (88–100)	100 (72–100)
Dose 75				
AD3, AD5, FBP				
(Reader 1–3)	100 (88–100)	100 (72–100)	100 (88–100)	100 (72–100)

Dose 25, 50, and 75 indicates the percentage of the original dose values; AD3 = ADMIRE 3; AD5 = ADMIRE 5; FBP = filtered back projection; CI, confidence interval; PPV, positive predicted value; NPV, negative predicted value.
